# Supplementary figures and images for: Circulating resistin and follistatin levels in obese and non-obese women with polycystic ovary syndrome: A systematic review and meta-analysis
Source: PLoS One. 2021 Mar 19;16(3):e0246200. doi: 10.1371/journal.pone.0246200 (PMC7978365; doi:10.1371/journal.pone.0246200)

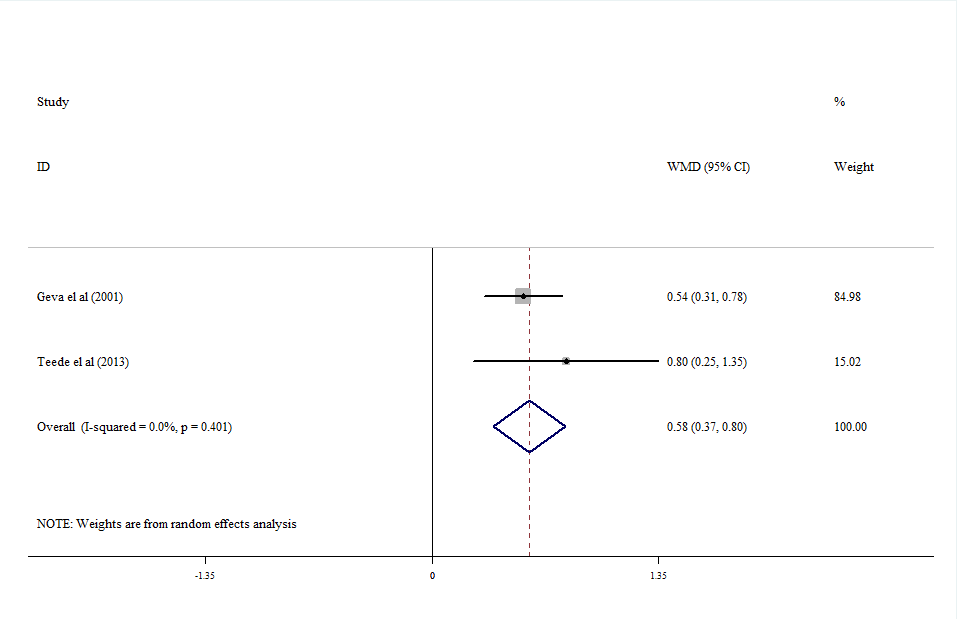


S2 Fig. Forest plot for the circulating follistatin in obese PCOS women vs. obese controls.

Supplement: S2 Fig — (DOCX) [file pone.0246200.s003.docx]
