# Supplementary material for: Circulating resistin and follistatin levels in obese and non-obese women with polycystic ovary syndrome: A systematic review and meta-analysis
Source: PLoS One. 2021 Mar 19;16(3):e0246200. doi: 10.1371/journal.pone.0246200 (PMC7978365; doi:10.1371/journal.pone.0246200)
Supplement: S3 Fig — (DOCX) [file pone.0246200.s004.docx]

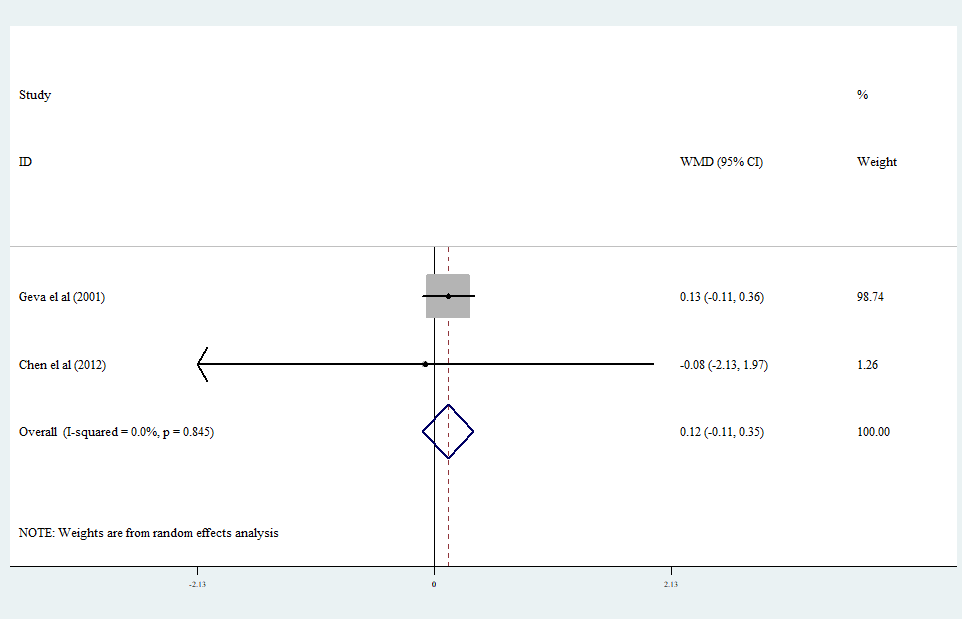


S3 Fig. Forest plot for the circulating follistatin in obese PCOS compared with non-obese PCOS women.
