# Supplementary material for: Circulating resistin and follistatin levels in obese and non-obese women with polycystic ovary syndrome: A systematic review and meta-analysis
Source: PLoS One. 2021 Mar 19;16(3):e0246200. doi: 10.1371/journal.pone.0246200 (PMC7978365; doi:10.1371/journal.pone.0246200)
Supplement: S1 Table — (DOCX) [file pone.0246200.s005.docx]

**S1 Table.** Quality assessment of studies included in this systematic review and meta-analysis according to the Newcastle-Ottawa Scale (NOS) criteria

|  | Selection of case and controls | | | | Comparability | Exposure | | | Total score |
| --- | --- | --- | --- | --- | --- | --- | --- | --- | --- |
|  | **Is the Case Definition Adequate** | **Representativeness of the Cases** | **Selection of Controls** | **Definition of Controls** | **Comparability of cases and controls on the basis of the design or analysis** | **Ascertainment of exposure** | **Same method of ascertainment for cases and controls** | **Non Response Rate** | **9** |
| Panidis el al. 2004 | * | NA | NA | * | * | * | * | NA | 5 |
| Seow el al. 2004 | * | NA | NA | * | ** | * | * | NA | 6 |
| LU el al. 2005 | * | NA | NA | * | * | * | * | NA | 5 |
| Seow el al. 2005 | * | * | NA | * | ** | * | * | NA | 7 |
| Morreale el al. 2006 | * | * | * | * | * | * | * | NA | 8 |
| Bideci el al. 2008 | * | NA | NA | * | * | * | * | NA | 5 |
| YILMAZ et al. 2009 | * | NA | NA | * | NA | * | * | NA | 4 |
| ARIKAN et al. 2010 | * | NA | NA | * | ** | * | * | NA | 6 |
| Glinianowicz el al. 2011 | * | NA | NA | * | * | * | * | NA | 5 |
| Glinianowicz el al. 2013 | * | NA | NA | * | * | * | * | NA | 5 |
| Cassar el al. 2015 | * | * | * | * | * | * | * | NA | 7 |
| Oz Gul el al. 2015 | * | NA | NA | * | * | * | * | NA | 5 |
| Nambiar el al. 2016 | * | NA | NA | * | NA | * | * | NA | 4 |
| CHEN el al. 2007 | * | NA | NA | * | NA | * | * | NA | 4 |
| CHU el al. 2009 | * | NA | NA | * | * | * | * | NA | 5 |
| WANG el al. 2010 | * | NA | NA | * | * | * | * | NA | 5 |
| Sarray el al. 2015 | * | NA | NA | * | ** | * | * | NA | 6 |
| Yasar NAWAZ. 2020 | * | NA | NA | * | ** | * | * | NA | 6 |
| Bertha Pangaribuan. 2011 | * | NA | NA | * | * | * | * | NA | 5 |
| GUVEN et al. 2010 | * | NA | NA | * | ** | * | * | NA | 6 |
| M.Erkan et al. 2014 | * | * | * | * | NA | * | * | NA | 6 |
| Christian Obirikorang. 2019 | * | * | * | * | NA | * | * | NA | 6 |
| Nikolaos Spanos. 2012 | * | NA | NA | * | * | * | * | NA | 5 |
| Baldani et al. 2019 | * | NA | NA | * | ** | * | * | NA | 6 |
| Behboudi‑Gandevani, 2017 | * | NA | NA | * | NA | * | * | NA | 4 |
| Mohd Ashraf Ganie, 2019 | * | * | * | * | ** | * | * | NA | 8 |
| Farshchian, 2014 | * | NA | NA | * | ** | * | * | NA | 6 |
| Atheer Mahde, 2009 | * | NA | NA | * | ** | * | * | NA | 6 |
| Nadine M. P. Daan, 2016 | * | NA | NA | * | NA | * | * | NA | 4 |
| Korczala, 2008 | * | * | * | * | NA | * | * | NA | 6 |
| Hung Shen, 2015 | * | NA | NA | * | * | * | * | NA | 5 |
| Geva el al, 2001 | * | NA | NA | * | NA | * | * | NA | 4 |
| Chen el al. 2012 | * | NA | NA | * | NA | * | * | NA | 4 |
| Teede el al. 2013 | * | * | * | * | NA | * | * | NA | 6 |
| Norman el al., 2001 | * | NA | NA | * | NA | * | * | NA | 4 |
| Shen el al. 2004 | NA | NA | NA | * | NA | * | * | NA | 3 |
| Chen el al. 2009 | * | NA | NA | * | NA | * | * | NA | 4 |
| Köninger, 2018 | * | * | * | * | NA | * | * | NA | 6 |
| Adnan Kensara, 2018 | * | * | * | * | ** | * | * | NA | 8 |
| Baranova, 2013 | * | NA | NA | * | NA | * | * | NA | 4 |
| Capoglu, 2009 | * | NA | NA | * | ** | * | * | NA | 6 |
| Carmina, 2005 | * | NA | * | * | ** | * | * | NA | 7 |
| DİKMEN, 2010 | * | NA | * | * | ** | * | * | NA | 7 |
| WANG, 2012 | * | NA | * | * | NA | * | * | NA | 5 |
| Yılmaz, 2005 | * | NA | * | * | ** | * | * | NA | 7 |
| Munir, 2005 | * | NA | NA | * | * | * | * | NA | 5 |
| Suganthi, 2010 | * | NA | * | * | NA | * | * | NA | 5 |

NA: Not Applicable
