# Supplementary material for: Circulating resistin and follistatin levels in obese and non-obese women with polycystic ovary syndrome: A systematic review and meta-analysis
Source: PLoS One. 2021 Mar 19;16(3):e0246200. doi: 10.1371/journal.pone.0246200 (PMC7978365; doi:10.1371/journal.pone.0246200)
Supplement: S2 Table — (DOCX) [file pone.0246200.s006.docx]

S2 Table. Sensitivity analysis by omitting single studies for studies investigating resistin levels in PCOS compared with healthy control women.

|  | 95% Confidence interval (CI) | | |
| --- | --- | --- | --- |
| Study omitted | WMD | Lower limit | Upper limit |
| Seow el al (2004) | 1.99 | 1.19 | 2.79 |
| LU el al (2005) | 1.95 | 1.23 | 2.66 |
| Seow el al (2005) | 1.97 | 1.26 | 2.68 |
| Morreale el al (2006) | 1.97 | 1.25 | 2.69 |
| Bideci el al (2008) | 1.98 | 1.27 | 2.69 |
| ARIKAN (2010) | 1.99 | 1.27 | 2.71 |
| Glinianowicz el al (2011) | 1.98 | 1.27 | 2.70 |
| Glinianowicz el al (2013) | 2.02 | 1.29 | 2.75 |
| Cassar el al (2015) | 2.00 | 1.24 | 2.77 |
| Oz Gul el al (2015) | 2.00 | 1.29 | 2.72 |
| Nambiar el al (2016) | 1.89 | 1.19 | 2.59 |
| CHEN el al (2007) | 2.08 | 1.37 | 2.80 |
| CHU el al (2009) | 1.87 | 1.15 | 2.58 |
| WANG el al (2010) | 1.88 | 1.16 | 2.60 |
| Sarray el al (2015) | 1.94 | 1.21 | 2.67 |
| Yasar NAWAZ (2020) | 1.83 | 1.12 | 2.54 |
| Bertha Pangaribuan (2011) | 2.00 | 1.28 | 2.72 |
| GUVEN (2010) | 2.04 | 1.33 | 2.75 |
| M.Erkan (2014) | 1.99 | 1.27 | 2.71 |
| Christian Obirikorang (2019) | 2.00 | 1.28 | 2.71 |
| Nikolaos Spanos (2012) | 2.03 | 1.30 | 2.760 |
| Baldani (2019) | 1.92 | 1.20 | 2.64 |
| Behboudi-Gandevani (2017) | 2.00 | 1.25 | 2.75 |
| Mohd Ashraf Ganie (2019) | 1.99 | 1.26 | 2.72 |
| Farshchian (2014) | 2.00 | 1.28 | 2.73 |
| Atheer Mahde (2009) | 1.83 | 1.17 | 2.48 |
| Dimitrios Panidis (2004) | 1.89 | 1.18 | 2.60 |
| Nadine M. P. Daan (2016) | 1.95 | 1.23 | 2.66 |
| Nadine M. P. Daan (2016) | 2.02 | 1.31 | 2.74 |
| Korczala (2008) | 1.95 | 1.23 | 2.68 |
| Hung Shen (2015) | 2.06 | 1.34 | 2.78 |
| YILMAZ (2009) | 1.75 | 1.05 | 2.45 |
| Baranova (2013) | 2.04 | 1.33 | 2.76 |
| Çapoglu (2009) | 1.89 | 1.17 | 2.60 |
| Carmina (2005) | 1.98 | 1.26 | 2.71 |
| DIKMEN (2010) | 2.01 | 1.23 | 2.78 |
| WANG (2012) | 1.64 | 1.01 | 2.33 |
| Yilmaz (2005) | 1.93 | 1.21 | 2.65 |
| Munir (2005) | 1.87 | 1.16 | 2.59 |
| **Combined** | **1.96** | **1.25** | **2.67** |
