# Supplementary material for: Circulating resistin and follistatin levels in obese and non-obese women with polycystic ovary syndrome: A systematic review and meta-analysis
Source: PLoS One. 2021 Mar 19;16(3):e0246200. doi: 10.1371/journal.pone.0246200 (PMC7978365; doi:10.1371/journal.pone.0246200)
Supplement: S3 Table — (DOCX) [file pone.0246200.s007.docx]

S3 Table. Sensitivity analysis by omitting single studies for studies investigating follistatin levels in PCOS compared with healthy control women.

|  | | 95% Confidence interval (CI) | |
| --- | --- | --- | --- |
| Study omitted | WMD | Lower limit | Upper limit |
| Geva el al (2001) | 0.43 | 0.29 | 0.56 |
| Chen el al (2012) | 0.46 | 0.31 | 0.62 |
| Teede el al (2013) | 0.39 | 0.24 | 0.53 |
| Norman el al (2001) | 0.48 | 0.32 | 0.64 |
| Shen el al (2004) | 0.43 | 0.27 | 0.58 |
| Chen el al (2009) | 0.38 | 0.23 | 0.52 |
| Köninger (2018) | 0.46 | 0.31 | 0.61 |
| Adnan Kensara (2018) | 0.42 | 0.28 | 0.57 |
| **Combined** | **0.43** | **29** | **0.58** |
