# Supplementary material for: Circulating resistin and follistatin levels in obese and non-obese women with polycystic ovary syndrome: A systematic review and meta-analysis
Source: PLoS One. 2021 Mar 19;16(3):e0246200. doi: 10.1371/journal.pone.0246200 (PMC7978365; doi:10.1371/journal.pone.0246200)
Supplement: Attachment — Submitted filename: response letter (1).docx [file pone.0246200.s009.docx]

**12 January 2021**

Re:

MS title: " **Circulating resistin and follistatin levels in obese and non-obese women with polycystic ovary syndrome: a systematic review and meta-analysis (Ms#
PONE-D-20-19835)"**

**Dear Editor-in-Chief of PLOS ONE**

We appreciate the time and efforts by the editor and referees in reviewing our manuscript. Below are the reviewers’ verbatim comments. All changes in the manuscript have been highlighted with red font.

We hope that with these changes and clarifications, our manuscript will be acceptable for publication. Nevertheless, we are prepared to revise our manuscript further, should it be necessary.

**COMMENTS FOR THE AUTHOR:**

Reviewer #2: Line 137, “Publication bias was evaluated using funnel plot…”, but no funnel plots were provided. Please add funnel plots for those meta-analysis of 10 or more studies.

**Response: funnel plots for meta-analysis of 10 or more studies were added (see Fig 8)**

Yours sincerely,

**Shahab Alizadeh, PhD.**

**Department of Clinical Nutrition**

**School of Nutritional Sciences and Dietetics**

**Tehran University of Medical Sciences**

**Fax: +98-21-889 559 79, Telephone: +98-9197604090**

**Email address:** [**sh_alizadeh@razi.tums.ac.ir**](mailto:%20sh_alizadeh@razi.tums.ac.ir%20)
